# Ophthalmic In Situ Nanocomposite Gel for Delivery of a Hydrophobic Antioxidant

**DOI:** 10.3390/gels11020105

**Published:** 2025-02-02

**Authors:** Marta Slavkova, Christina Voycheva, Teodora Popova, Borislav Tzankov, Diana Tzankova, Ivanka Spassova, Daniela Kovacheva, Denitsa Stefanova, Virginia Tzankova, Krassimira Yoncheva

**Affiliations:** 1 Department of Pharmaceutical Technology and Biopharmacy, Faculty of Pharmacy, Medical University of Sofia, 1000 Sofia, Bulgaria; hvoycheva@pharmfac.mu-sofia.bg (C.V.); tpopova@pharmfac.mu-sofia.bg (T.P.); btzankov@pharmfac.mu-sofia.bg (B.T.); kyoncheva@pharmfac.mu-sofia.bg (K.Y.); 2 Department of Pharmaceutical Chemistry, Faculty of Pharmacy, Medical University of Sofia, 1000 Sofia, Bulgaria; d.tsankova@pharmfac.mu-sofia.bg; 3 Institute of General and Inorganic Chemistry, Bulgarian Academy of Sciences, 1113 Sofia, Bulgaria; ispasova@svr.igic.bas.bg (I.S.); didka@svr.igic.bas.bg (D.K.); 4 Department of Pharmacology, Pharmacotherapy and Toxicology, Faculty of Pharmacy, Medical University of Sofia, 1000 Sofia, Bulgaria; denitsa.stefanova@pharmfac.mu-sofia.bg (D.S.); vtzankova@pharmfac.mu-sofia.bg (V.T.)

**Keywords:** composite hydrogel, poly-(lactic-co-glycolic acid) PLGA nanoparticles, curcumin, ophthalmic, HaCaT

## Abstract

The topical administration of in situ hydrogels for ocular pathologies is a promising application strategy for providing high effectiveness and patient compliance. Curcumin, a natural polyphenol, possesses all the prerequisites for successful therapy of ophthalmic diseases, but unfortunately its physicochemical properties hurdle the practical use. Applying a composite in situ thermoresponsive hydrogel formulation embedded with polymer nanoparticles is a potent strategy to overcome all the identified drawbacks. In the present work we prepared uniform spherical nanoparticles (296.4 ± 3.1 nm) efficiently loaded with curcumin (EE% 82.5 ± 2.3%) based on the biocompatible and biodegradable poly-(lactic-co-glycolic acid). They were thoroughly physicochemically characterized in terms of FTIR, SEM, TGA, and DLS, in vitro release following Fickian diffusion (45.62 ± 2.37%), and stability over 6 months. Their lack of cytotoxicity was demonstrated in vitro on HaCaT cell lines, and the potential for antioxidant protection was also outlined, starting from concentrations as low as 0.1 µM and reaching 41% protection at 5 µM. An in situ thermoresponsive hydrogel (17% *w*/*v* poloxamer 407 and 0.1% Carbopol) with suitable properties for ophthalmic application was optimized with respect to gelation temperature (31.40 ± 0.36 °C), gelling time (8.99 ± 0.28 s) upon tears dilution, and gel erosion (90.75 ± 4.06%). Upon curcumin-loaded nanoparticle embedding, the in situ hydrogels demonstrated appropriate pseudoplastic behavior and viscosity at 35 °C (2129 ± 24 Pa∙s), 6-fold increase in the permeation, and prolonged release over 6 h.

## 1. Introduction

The scientific interest in recent years has been aimed towards active constituents from natural origins due to their versatility and minor toxicity effects. The isolation, characterization, and dosage form preparation, together with pharmacological and toxicological evaluation of phytochemicals, experience a renaissance in the pharmaceutical field [1]. Their pleiotropic effects could lead to significant health benefits. Such a natural compound is curcumin (CRC), which is a non-flavonoid polyphenol isolated from the rhizome of *Curcuma longa* L. It is known to possess anti-inflammatory, antioxidant, antimicrobial, anticancer, antiapoptotic, and immunoregulatory effects [2]. Curcumin is a scientifically promising compound due to its wide therapeutic potential together with its typical yellow/green fluorescence that contribute to its theranostic application [3]. Moreover, it could be beneficial for the therapy of numerous ocular pathologies associated with oxidative stress, like dry eye, cataract, glaucoma, macular degeneration, etc., due to its antioxidant activity and other pharmacological effects [4,5]. However, curcumin’s practical application is limited by its low bioavailability regardless of the route of administration [6], and hence extensive effort has been concentrated in resolving this issue to provide practical treatment translation. Nanotechnology is the most commonly considered opportunity because of its versatility through wide variety of nanocarriers [7,8]. The polymeric nanoparticles, and especially those composed of biocompatible and biodegradable polymers, have been extensively studied for the delivery of different drugs and routes of administration [9]. Poly-(lactic-co-glycolic acid) (PLGA) is a polymer considered by the Food and Drug Administration (FDA) as suitable for biomedical application due to its limited toxicity and established biodegradation [10]. There are several studies presenting the ability of PLGA to overcome some of the limitations of curcumin, which are comprehensively outlined in the light of anticancer application in a review by Feltrin et al. [11].

The topical instillation is the preferable way of application for ocular pathologies since it offers convenience and non-invasiveness. Nevertheless, the eye is characterized by static (tight conjunctival junctions) and dynamic barriers (tear film turnover, eyelid movements) that significantly reduce the bioavailability (less than 5%) of any drug applied [12] and further diminishes the possibility for curcumin effectiveness in ophthalmology. Even though nanoparticles gain some success in overcoming most of the drawbacks, there is still the issue with providing sufficient contact time with the eye surface [13]. The pre-corneal drug clearance would also have a negative impact on curcumin’s effectiveness. An elucidation of this difficulty could be the use of a semisolid formulation such as hydrogel. Hydrogels are characterized by a three-dimensional structure and the capacity to absorb significant quantities of water. The stimuli-responsive hydrogels are easily applied in situ gels that further contribute to enhancing effectiveness by furnishing better and long-lasting contact as well as providing prolonged drug release. Among the available stimuli, temperature is the least irritant one and most preferable trigger for sol–gel transition for the in situ ocular hydrogels [13,14]. In order to improve gelling properties, rheology, and/or adhesion, a combination of temperature-sensitive polymers, acting as gelling agents, is usually applied [14]. Poloxamer 407 with a concentration ranging between 16% and 22% is a commonly applied thermosensitive synthetic polymer. It is an amphiphilic block copolymer with the ability to form micelles and thus improve the solubility of drugs such as curcumin [15]. Carbomer has been reported to be applied in combination with poloxamer 407 in order to strengthen the gel and enrich the in situ gelling system with bioadhesive properties [16,17,18]. Moreover, there are data demonstrating that carbomer can also act as a penetration enhancer [19]. Even though systems with curcumin loading within PLGA nanoparticles have been studied in the literature [20,21,22,23], their application in ophthalmology, together with the introduction of an optimal formulation, has not been fully addressed. In this regard, in the current study we aimed at developing curcumin-loaded PLGA nanoparticles within in situ thermoresponsive hydrogel for ocular delivery. Thorough physicochemical characterization of the nanoparticles, hydrogel optimization, and in vitro cytotoxicity assessment on the HaCaT cell line were performed. Keratinocytes are actively involved in the processes of epidermal repair and the protection of cellular immunity through the secretion of growth factors, cytokines, etc. The protective effect of curcumin loaded into nanoscale drug delivery systems based on poly-(lactic-co-glycolic acid) (PLGA) was investigated in a cellular model of oxidative stress induced by H_2_O_2_, utilizing the HaCaT cell line, which originates from human keratinocytes. HaCaT cells produce cytokines, such as IL-10, IL-1β, IL-6, IL-8, IL-12p70, and TNF-α, which are major modulators of the inflammatory response. IL-1β has been found to be an established biomarker of heavy keratinocyte injury [24].

## 2. Results and Discussion

### 2.1. Nanoparticles Preparation and Characterization

The polymer nanoparticles based on poly-(lactic-co-glycolic acid) (PLGA) were successfully prepared by the solvent–evaporation method. The empty nanoparticles were labeled NP and the curcumin-loaded ones NP-CRC. The curcumin loading showed to be successful with 82.5 ± 2.3% encapsulation efficiency, which is typical for PLGA nanoparticles [23]. The results presented in Table 1 showed the formation of relatively small nanoparticles with uniform size distribution and negative zeta potential. The loading of curcumin results in a narrower size distribution and a drop in the absolute value of zeta potential due to possible hydrogen interactions and modification of the structure of the nanoparticles. In the literature, very different zeta potential values for PLGA-based nanoparticles loaded with curcumin can be found. However, a tendency is observed that when dichloromethane is used as an organic solvent during the preparation, the absolute value of zeta potential is usually below 10 mV [11].

The nanoparticles’ size is an important characteristic rendering some of their most important properties, such as improved dissolution and bioavailability of drugs with low aqueous solubility, such as curcumin. The optimal parameters depend on the intended route of application. In the case of ocular delivery, it is stated that sizes of less than 400 nm could overcome the barrier functions of the cornea [25,26]. The nanoparticles presented here fall in this range with sizes around 300 nm in the suspension form immediately after preparation (Table 1). Similar results were presented by other studies [10], although some differences can also be observed, which could be attributed to the different types of PLGA and polyvinyl alcohol (PVA) used. The negative charge of the nanoparticles in the present study is most probably due to the presence of carboxyl groups from the PLGA, which are partially covered by PVA, and thus values of about −15 mV are observed. Such assumption could be found in other studies [10,23,27]. Covering of the carboxyl groups on the nanoparticles’ surface is the possible reason for the drop in the absolute value of zeta potential after curcumin loading. Typically, zeta potentials higher than ±30 mV are considered optimal regarding nanoparticles’ stability [27] and can affect the adhesive properties on the negatively charged ocular surface [28]. Thus, it can be expected that the prepared nanoparticles would possess suitable properties for eye delivery of curcumin.

The colloidal stability of the nanoparticles was evaluated in suspension and after lyophilization with cryoprotectant (mannitol) and storage at 4 °C. The results (Table 1) showed that the nanoparticles, especially the NP-CRC, when stored as a suspension, increased in size even though still comparably uniform. The empty NP had also significant size increase. This observed difference could be attributed to the decrease in the zeta potential, which is more obvious in the NP-CRC. After 6 months of storage, the zeta of all suspension samples was decreased as an absolute value.

The lyophilization in the presence of 5% (*w*/*v*) mannitol as a cryoprotectant was successful in preserving their characteristics as opposed to the nanoparticles stored in suspension. This observation is supposed to be a result of the formation of a steric barrier in the crystal state and thus preventing the nanoparticles’ aggregation during freeze-drying [10,29]. The proposed mechanism of mannitol cryostabilization is based on its crystallization during freezing and therefore providing sufficient distance for the nanoparticles to prevent Van der Waals interactions between them, which in turn would avert an increase in particle size. This is in accordance with the PXRD results. The addition of cryoprotectants leads to further formation of hydrogen bonds, and a reduction in zeta potential is observed [29]. The observed decrease in particle size could be associated with the specific crystal formation of mannitol during the freezing period and the crystals’ growth, even though the exact reason for the observed phenomenon is still not fully understood [30]. Similar results regarding the decrease in PLGA nanoparticles’ size with the addition of cryoprotectant were previously reported [31]. During six months of storage, as expected, there was no significant change in particle size.

**Figure 1:**
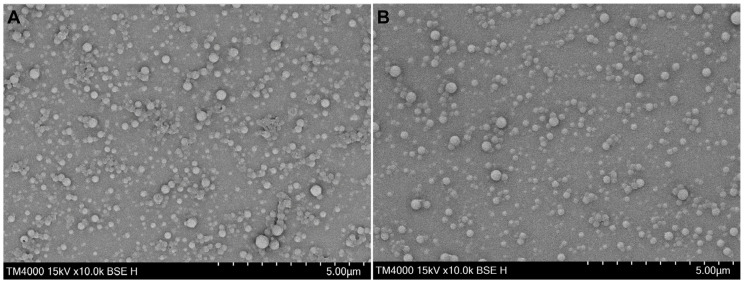
SEM images at a magnification 10,000× of plain NP (**A**) and NP-CRC (**B**).

The morphology of the prepared polymeric nanoparticles immediately after their formation was evaluated with the help of SEM. The images given in Figure 1 present their spherical shape and relatively smooth surface while simultaneously confirming the findings of the DLS analysis. Some aggregates can be found, probably due to the sample preparation by drying at room temperature.

**Figure 2:**
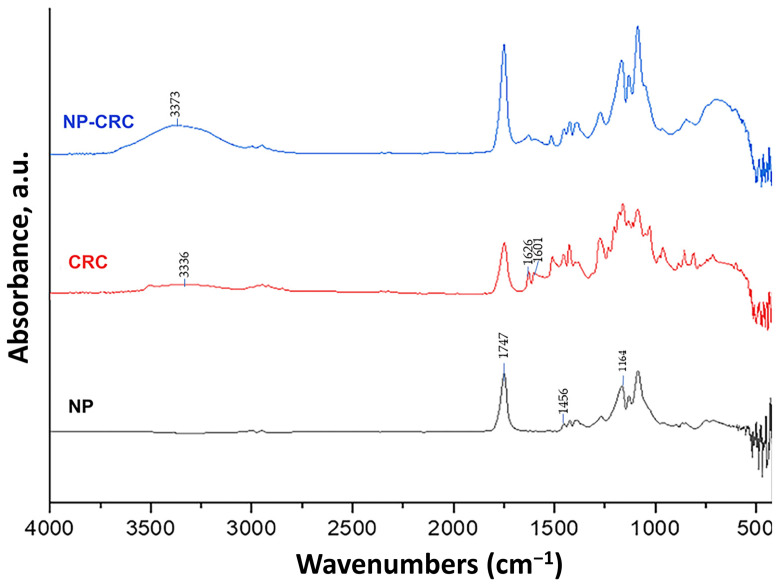
FTIR spectra of the plain NP, curcumin, and curcumin-loaded NP-CRC.

The Fourier-transform infrared (FTIR) analysis (Figure 2) showed that the plain NP were characterized by the absorption band associated with the stretching vibration of the carbonyl group at 1747 cm^−1^, the deformation motion of the C–H bonds within the O–CH_2_ moiety at 1456 cm^−1^, and C–O stretching (1164 cm^−1^) [32,33]. Curcumin showed characteristic absorption bands at 3336 cm^−1^ corresponding to the phenolic O-H stretching vibrations, 1626 cm^−1^ associated with the C=C stretching in the aromatic structure, 1601 cm^−1^ attributed to benzene ring vibrations, among others [34]. In the spectrum of NP-CRC, curcumin’s main absorption peaks are weakened, indicating that the curcumin molecules were predominantly encapsulated within the NP. The shift and enhancement of the CRC peak to 3373 cm^−1^ in the NP-CRC spectrum suggested an interaction of the phenolic –OH of curcumin with PLGA likely mediated by hydrogen bonding. This observation aligns with the observed decrease in the zeta potential upon drug loading.

**Figure 3:**
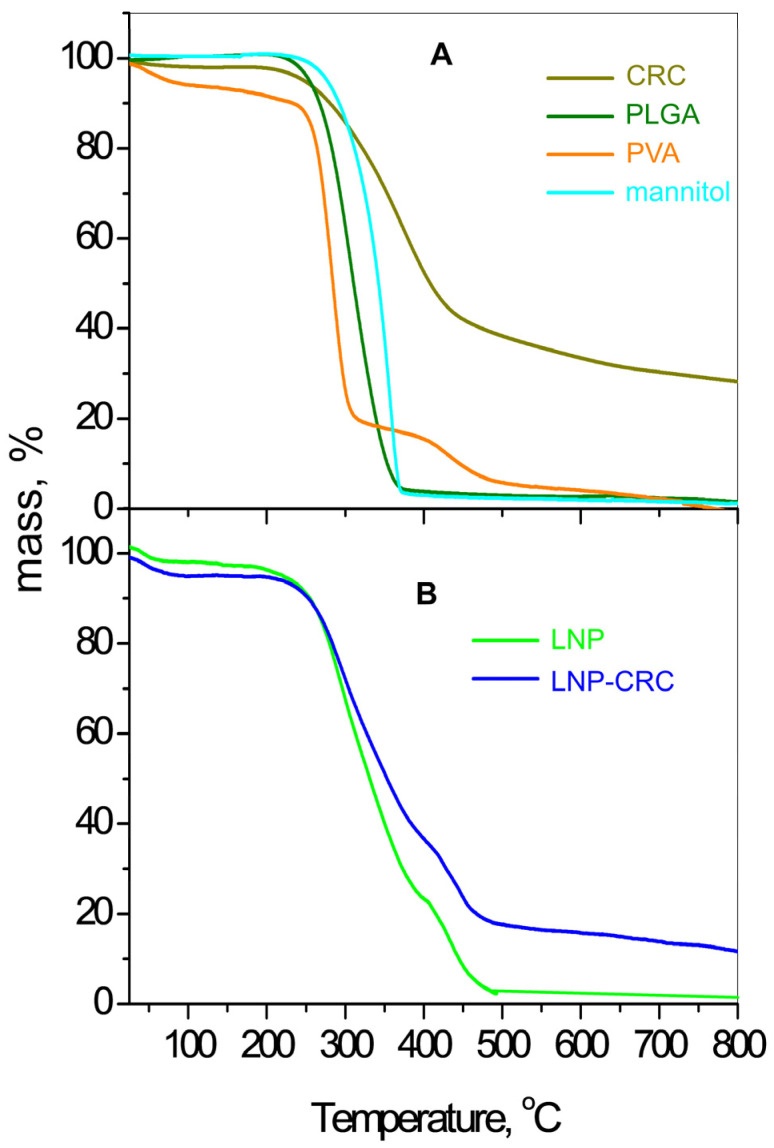
TG profiles of the initial materials (**A**); plain nanoparticle LNP and curcumin-loaded LNP-CRC (**B**).

The results from the thermogravimetric analyses are presented in Figure 3. The TG profiles of the initial constituents are placed in Figure 3A. The TG curve of PLGA is presented in one step from 174 to 495 °C, where complete degradation of the polymer occurs with a mass loss of about 98%. The thermal profile of polyvinyl alcohol (PVA) consists of four stages. The first stage includes a mass loss of 5% in the range of 20–100 °C, which is attributed to physically adsorbed water. The second stage with a mass loss of 75% is up to 350 °C and is due to partial removal of OH groups and polyene formation [35]. The third stage is at 350–500 °C with a mass loss of 5.5% and reflects low-mass oxygen-containing products, and the final step is attributed to carbonized residue. The thermal curve of the free curcumin shows no mass loss below 200 °C, and the mean loss of about 40% is registered from 200 to 400 °C. At higher temperatures, to the end of the analysis, the residue is 30%. This implies high thermal stability of the material. A complete 99% degradation of D-mannitol is registered from 230 to 380 °C. The thermal behavior of the freeze-dried empty nanoparticles and curcumin-loaded ones is presented in Figure 3B. Their thermal curves, as expected, resemble in their course those of PLGA and PVA as constituents of the nanoparticles. However, the addition of mannitol enhances the thermal stability of the empty nanoparticles with about 50 °C at the middle temperature range. The TG curve of LNP-CRC distinctly indicates the successful loading of the curcumin into the empty nanoparticles. Additional thermal stabilization due to the influence of the curcumin presence is clearly pronounced at temperatures above 300 °C for the lyophilized loaded particles.

**Figure 4:**
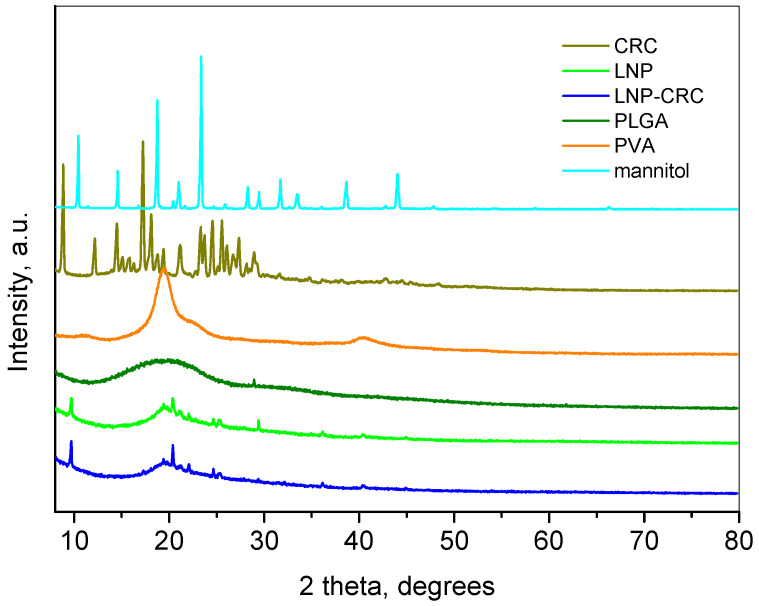
Powder XRD patterns of the initial compounds, lyophilized empty LNP, and curcumin-loaded nanoparticle LNP-CRC.

The PXRD pattern of free CRC (Figure 4) shows many intensive peaks with positions coinciding with the referent pattern of curcumin (ICDD-PDF # 00-009-0816). It crystallizes in monoclinic Space Group with the following unit cell parameters refined in the present study: a = 12.694(4) Å, b = 7.219(1) Å, c = 19.885(6) Å and β = 95.35(2)°, which are close to the parameters presented in the literature [36]. The PXRD pattern of mannitol also shows a high crystalline nature of this compound. Its pattern corresponds to the orthorhombic modification of 2-D mannitol (ICDD-PDF # 00-022-1797). The pattern of Polyvinyl alcohol consists of a few broad peaks reflecting its chain structure [37], which is described as monoclinic and the refined parameters are: a = 7.71(2) Å, b = 2.51(1) Å, c = 5.45(5) Å, β = 90.4(5)°. The diffraction pattern of PLGA represents an amorphous hump at about 20°2θ.

The empty (LNP) and loaded (LNP-CRC) freeze-dried samples show patterns that resemble a combination of those of PLGA and PVA. The major part of the sharp peaks appearing on the patterns are attributed to the presence of mannitol as a stabilizing agent. It is worth mentioning that the structure of mannitol is changed to a monoclinic one upon the freeze-drying procedure and can be related to the ICDD-PDF # 00-022-1794. Several peaks in the LNP-CRC pattern can be assigned to the presence of crystalline curcumin, evidencing its successful loading.

**Figure 5:**
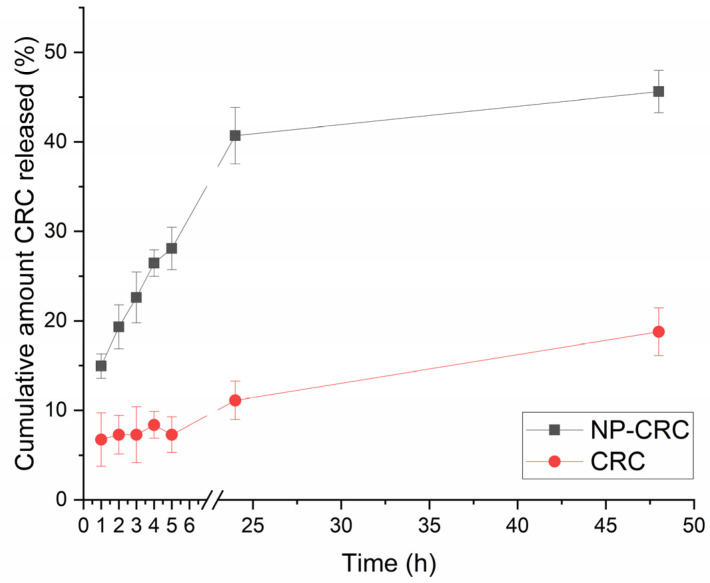
In vitro dissolution profile of the free CRC and loaded nanoparticle NP-CRC. The results present the mean of three replicates with the SD.

The in vitro dissolution test revealed improved release of CRC from NP-CRC over time in comparison to the free drug (Figure 5). It is most likely due to the increased specific surface area in the case of nanoparticles. Similar results are reported in other articles as well [11,23,38]. The improved dissolution could be expected to be associated with improved efficacy of the drug. The fitting of the release data showed the highest correlation (R^2^ = 0.9625) for the Korsmeyer–Peppas model. This value of the release exponent (n = 0.283) suggests that the predominant release mechanism is Fickian diffusion. These findings are in agreement with previous studies [39,40].

### 2.2. In Vitro Cytotoxicity and Antioxidant Protection Studies

#### 2.2.1. Cytotoxicity Study on Human HaCaT Cell Line

The toxicological evaluation of drug delivery systems is an important step in their preclinical safety testing. Ultrafine particles and nanoparticles can cause a variety of toxic responses that are heavily influenced by their physicochemical properties [41]. Toxic side-effects such as airway inflammation, oxidative stress, and distal organ involvement have been identified following inhalation or implantation of ultrafine particles in the lungs of experimental animals. In vitro studies in cell culture have also shown a propensity towards causing oxidative stress and increased concentrations of regulators of inflammation and apoptosis regulators in response to the application of ultrafine particles [42].

**Figure 6:**
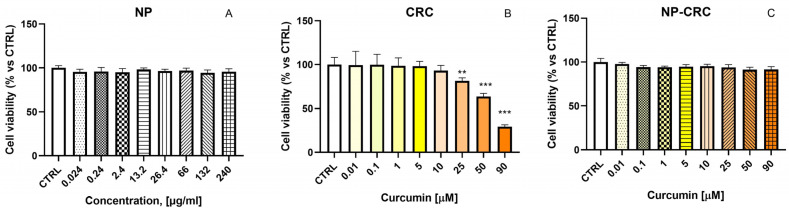
In vitro cytotoxicity assessment in human keratinocytes HaCaT, treated with the non-loaded nanoscale drug delivery system (NP) at 0.024–240 μg/mL (**A**), free curcumin (CRC) (**B**), and nanoparticle-loaded curcumin (NP-CRC) (**C**) at 0.01–90 μM. Data are presented as mean ± SD. Statistical analysis was performed using ANOVA with Dunnett’s post-test ** *p* < 0.01; *** *p* < 0.001.

To evaluate the safety of the proposed nanocarrier, the effects of non-loaded nanoparticles (0.024–240 μg/mL) on HaCaT cells were first evaluated (Figure 6A). HaCaT cell line is a suitable model system for studying adverse effects on the epithelium, including studies of ocular toxicity [43].

CRC showed no cytotoxicity at concentrations up to 10 μM. In contrast, statistically significant, concentration-dependent cytotoxicity was observed at higher concentrations (25–90 μM). At the highest treatment concentration of 90 μM, a decrease in cell vitality by 70% was observed (Figure 6B). Of interest are the data on the effects of CRC loading into the nanoparticles. In contrast to CRC, NP-CRC studied at equimolar concentrations showed no cytotoxic effects in HaCaT cells, even at the concentrations of 25–90 μM (Figure 6C).

#### 2.2.2. In Vitro Protection by Curcumin-Loaded Nanoparticles in H_2_O_2_-Induced Oxidative Stress Model

We evaluated the defense effects of NP-CRC in a model of H_2_O_2_-induced oxidative stress in HaCaT cells. The process of H_2_O_2_-induced cell damage involves a generation of reactive hydroxyl radicals and other species (Fenton reaction) that can further damage cellular proteins, lipids, and DNA. Epidermal cells are the target for oxidative damage, produced by toxic substances through the presented mechanism. If the increased amount of reactive oxygen species is not cleared by cellular antioxidant defense systems, this results in oxidative stress. Therefore, increasing the cellular antioxidant defenses is of great importance for preventing damage and maintaining normal cellular functions during oxidative stress.

**Figure 7:**
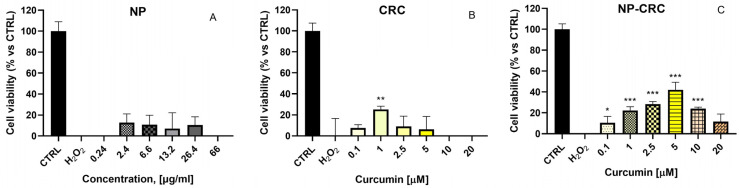
Protective effects of the (**A**) non-loaded nanoscale drug delivery system (NP) at 0.24–66 μM, (**B**) free curcumin (CRC), and (**C**) nanoparticle-loaded curcumin (NP-CRC) at 0.1–20 μM in a model of H_2_O_2_-induced damage in human keratinocyte-derived HaCaT cells. Data are presented as mean ± SD. Statistical analysis was performed using ANOVA with Dunnett’s post-test * *p* < 0.05; ** *p* < 0.01; *** *p* < 0.001.

Treatment of HaCaT cells with H_2_O_2_ (200 μM, 24 h) resulted in a statistically significant reduction in cell vitality by 40% compared to untreated controls. Non-loaded nanoparticles (NP) (0.24–66 μg/mL) did not cause statistically significant protection in the oxidative damage model (Figure 7A). In contrast, the preincubation of cells with CRC (1 μM) induced statistically significant protection (Figure 7B). The observed protective effects were statistically significant (** *p* < 0.01) and reached 25% versus the negative controls. Pretreatment of cells with NP-CRC exerted pronounced, statistically significant (* *p* < 0.05, *** *p* < 0.001) cytoprotective effects against H_2_O_2_-induced oxidative damage at the following concentrations: 0.1 μM, 1 μM, 2.5 μM, 5 μM, and 10 μM by 11%, 22%, 28%, 41%, and 24%, respectively. Noteworthy, in this model of oxidative stress, the protection caused by the loaded NP-CRC is more pronounced compared to the free, non-loaded CRC (Figure 7C). Preservation of cellular vitality was observed even at the lowest concentration studied, 0.1 μM. At this concentration, the free CRC did not exhibit protective effects.

### 2.3. Thermoresponsive In Situ Hydrogel Optimization

The in situ gels were based on the typical thermoresponsive polymer poloxamer 407, which is used in concertations ranging from 16% to 22% *w*/*v* to render suitable gelation temperature. The higher concentrations are associated with possible irritation [14], and thus in the present work the investigated diapason was set up to 18% *w*/*v* (Table 2). The Carbopol was chosen as a second gelling agent based on its known bioadhesive properties [17,18]. The concentrations were selected based on previous research stating that concentrations of 0.1% and 0.2% provide sufficient mucoadhesive properties without significantly affecting the liquid state of the gel at room temperature [16]. Furthermore, concentrations higher than 1% *w*/*v* in some cases could lead to loss of the thermoresponsive gelling capacity [17] or above 0.3% cause ocular irritation due to increased acidity [18].

The ocular surface temperature is stated to be variable in the interval of 31 °C to 37 °C between individuals, during different blinking times, and depends on the environmental conditions as well [44]. In the case of eye disease, it can be higher (e.g., in glaucoma [45]) or lower (e.g., in dry eye [46]). Having in mind the versatile properties of curcumin and its therapeutic potential for a variety of ophthalmic pathologies, an in situ gel with a gelation temperature higher than 30 °C would be most appropriate. Some of the prepared in situ hydrogels in the current work offer suitable gelation temperature (Table 2). The time of gelation upon dilution with simulated tear fluid (STF) is another important characteristic. The faster the gelation takes place, the lower the possibility would be for loss of formulation. Ideally, it should be less than 5 min, as the tear turnover is on average 15%/min [47]. Considering this, all of the proposed formulations seem suitable. Similar results have been observed in other studies [48,49].

**Figure 8:**
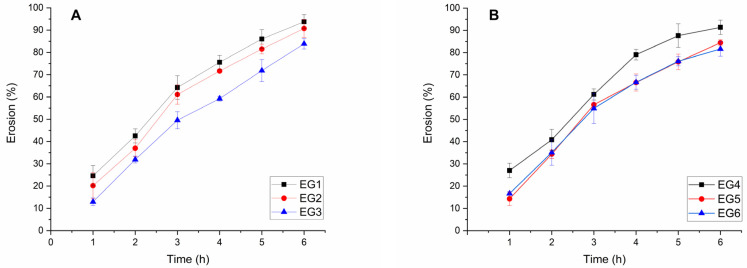
Erosion curve of the empty hydrogels with 0.1% (*w*/*v*) carbomer (**A**) and 0.2% (*w*/*v*) carbomer (**B**) over time in STF.

The gel erosion was also investigated after incubation with STF over time, and the erosion profiles were drawn (Figure 8). Generally, poloxamer hydrogels lack strength. Their porous, interpenetrating aqueous structure is responsible for the gel matrix erosion and is concentration dependent. Poloxamer 407 in concentrations less than 25% *w*/*v* fully erodes within 6 h [50]. The same observations could be made in the current study, and the higher the amount of poloxamer included, the lower the erosion rate is, even though the difference does not seem to be statistically significant (*p* = 0.69). The addition of a second gelling agent usually improves the gel strength [51] and thus reduces the gel erosion as observed here (Figure 8A,B). The one-way ANOVA analysis of the % eroded gel demonstrated that the difference is not significant (*p* = 0.93). Therefore, regarding the in vitro erosion of the gels, all samples render suitable for application as an ophthalmic delivery system due to the lack of expected nasolacrimal duct obstruction [52,53].

Based on all the results, formulation EG2 was selected as the optimal blank in situ hydrogel carrier. It possessed suitable gelation temperature and gelling time upon dilution with STF with adequate erosion over the studied period.

### 2.4. Composite In Situ Thermosensitive Gel for Curcumin Delivery

**Figure 9:**
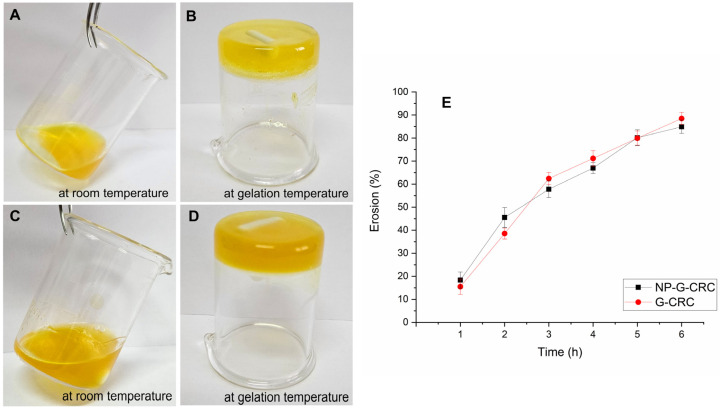
Visual appearance of the prepared G-CRC (**A**,**B**), NP-G-CRC (**C**,**D**), and their erosion profiles in STF (**E**).

The optimal gel formulation was further utilized for the embedding with curcumin-loaded PLGA nanoparticles and labeled as NP-G-CRC. For comparison purposes, the same hydrogel was loaded with free curcumin and coded G-CRC. Their physical appearance at room temperature and upon gelation is presented in Figure 9A–D. The thermoresponsive properties of the thus prepared drug-loaded gels were investigated in a similar manner as the empty gels. The CRC addition to the optimized gel formulation did not significantly affect its gelation temperature and gelling time (Table 3). The erosion profile revealed an almost identical pattern with 88.46% ± 2.7% eroded amount within 6 h as compared to 90.75% ± 4.06% in the case of the empty gel. The NP-CRC incorporation within the gel is associated with a slight decrease in the gelation temperature and time, even though not significant (Table 3). The erosion study revealed a slight fortification of the gel with 84.90% ± 2.9% loss of gel during the 6 h test period (Figure 9E). These results are in accordance with the rheology study and are expected due to the presence of nanoparticles in the hydrogel, which may interact with the cross-linked micellar structure of the poloxamer gel [54].

The rheology of the obtained G-CRC and NP-G-CRC gels was investigated at room temperature, mimicking the conditions of instillation at 35 °C (simulation of the eye surface temperature).

At room temperature (20 °C), the two samples were in liquid state and exhibited shear-dependent behavior—their viscosity decreased with increasing the shear rate. At a given shear rate (above 14 s^−1^), the values of dynamic viscosity became constant, and the viscosity of the nanocomposite hydrogel NP-G-CRC was higher than that of the free drug-loaded gel G-CRC (Table 3).

The pseudoplastic behavior of the applied hydrogel is desirable for mimicking the rheology of the natural tears. These gel properties would prolong the contact time during which the eye is being open, and at the same time, the ocular surface will be protected during blinking by the shear-thinning and reduction in viscosity since the applied formulation will be evenly spread [55,56]. The viscosity data presented here cover the minimum required values of 10 mPa to maintain sufficient precorneal residence [57].

At 35 °C the fluids transformed into gels. Thus, oscillation amplitude and frequency sweep tests were performed to demonstrate the main characteristics of the gels.

**Figure 10:**
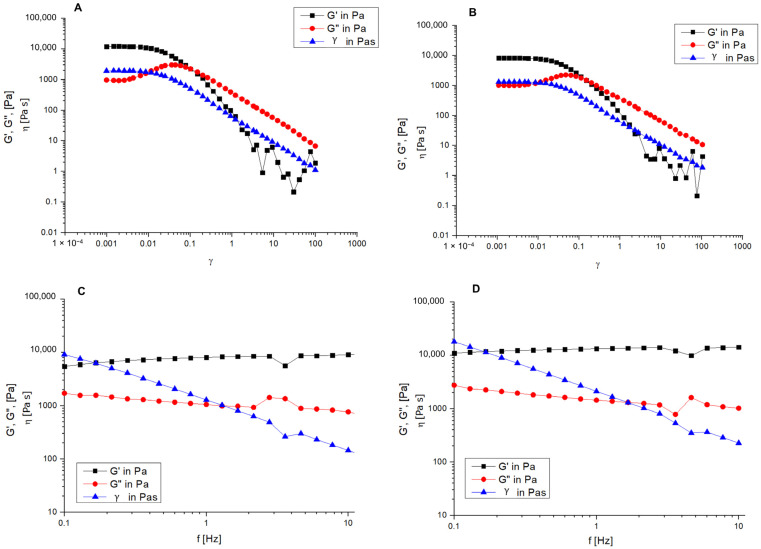
Oscillation amplitude test (**A**,**B**) and frequency sweep test (**C**,**D**) for the conventional hydrogel G-CRC (**A**,**C**) and the nanocomposite hydrogel NP-G-CRC (**B**,**D**) at 35 °C.

Dynamic rheological measurements revealed that the free drug-based gel (G-CRC) and nanocomposite gels (NP-G-CRC) have the typical physical gel behavior (Figure 10A,B). At very small strains, the samples are characterized with nearly constant G’ and G’’, where G’ >> G’’. In the initial area, the elastic component prevails. Increasing the strain is associated with some shear thickening, tailed by distinct shear thinning. The shear thinning is related to the disarrangement of the close-packed poloxamer micelles and the following micellar layers movement in the flow direction.

The frequency sweep test was carried out at a fixed stress value (0.01). It was based on the linear viscoelastic range determined earlier. All specimens can be considered a hard gel based on the results that the moduli are independent of the applied frequency and the elastic one considerably exceeds the viscous (G’ >> G’’) (Figure 10C,D). This is further supported by the lack of crossover of the curves in the studied frequency diapason.

Comparing the G’ value of the two gels (at a frequency of 1 Hz), we can conclude that the NP slightly reinforces the gel (8050 Pa for G-CRC and 13,300 Pa for NP-G-CRC).

**Figure 11:**
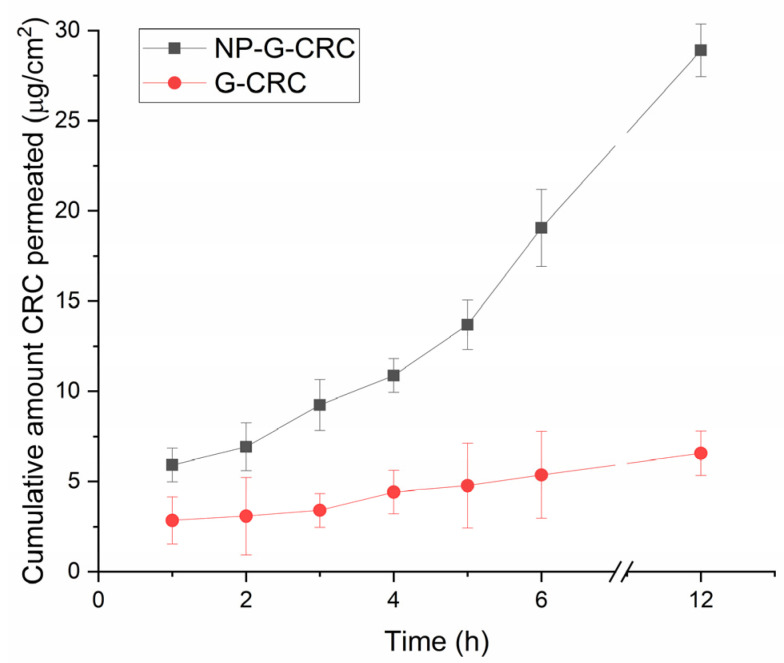
In vitro permeation profiles in STF in a Franz diffusion cell at 35 ± 0.5 °C; mean ± SD, n = 3.

The results from the in vitro permeation study with the two CRC-loaded hydrogels (CRC-G and NP-G-CRC) are presented in Figure 11. In both cases, the permeation is sustained, which is associated with the semisolid state of the hydrogel, which slows down the diffusion of the drug. In addition, the permeated CRC amount in the case of nanocomposite gel (NP-G-CRC) is higher than the one in the case of free drug loaded hydrogel (G-CRC). The embedding of nanoparticles within the hydrogel improves the drug penetration. It is associated most likely with the increased drug solubility.

There was a 6-fold increase in the flux observed (2.142 ± 0.125 µg/cm^2^.h as opposed to 0.355 ± 0.048 µg/cm^2^.h for the NP-G-CRC and G-CRC, correspondingly). Together with the suggested improved residence time on the ocular surface, this better permeation could be associated with ameliorated therapeutic efficacy of a very hydrophobic drug, curcumin.

## 3. Conclusions

In the present study, we outlined the successful development of PLGA-based nanocarriers and their loading with the hydrophobic natural polyphenol curcumin. Thorough physicochemical and biopharmaceutical characterization revealed suitable particle size, negative zeta potential, and improved electrophoretic as well as thermal stability. The in vitro cytotoxicity study performed in human HaCaT keratinocytes demonstrated that nanoscale drug delivery systems, based on poly-(lactic-co-glycolic acid) (PLGA), are not cytotoxic to HaCaT cells, which makes them a promising carrier for the delivery of hydrophobic active substances, such as curcumin. The increased antioxidant activity of curcumin-loaded PLGA nanoparticles could be the basis of further pharmacological and toxicological studies related to the possible application as protective agents against ocular toxicity caused by oxidative stress. A suitable semisolid nanocomposite carrier was optimized to provide acceptable topical application regarding its gelation temperature, gelling time, and erosion. The demonstrated pseudoplastic flow turned out optimal for easy application and prolonged residence time on the ocular surface. The in vitro permeation study was characterized by increased permeation through the synthetic cellulose membrane from the nanocomposite hydrogel.

In this work we present a thorough, wide-area, in vitro characterization of the PLGA-nanoparticles loaded with curcumin and their stability over time, which provides new insights into the applicability of curcumin as an antioxidant agent in ocular pathologies. Simultaneously, a promising dosage form for the practical eye application is investigated and thus improves the understanding of the effects of embedded nanoparticles on the gel’s properties and behavior. However, further studies could elucidate the actual in vivo potential of the proposed curcumin carrier system.

## 4. Materials and Methods

### 4.1. Materials

Curcumin (CRC), poly-(lactic-co-glycolic acid) (PLGA) with an average molecular weight of 20,000 (lactide:glycolide ratio 50:50), and methylene chloride were purchased from Sigma Aldrich Chemie GmbH (Steinheim, Germany). Polyvinyl alcohol (PVA) with a molecular weight of 22,000 was obtained from Fluka Chemie GmbH (Buchs, Switzerland). Kolliphor P 407 was purchased from BASF, Ludwigshafen, Germany. Carbopol Ultrez^TM^ 10 was sourced from Lubrizol, Brussels, Belgium. All other chemicals and reagents were of analytical grade and were utilized as received.

### 4.2. Preparation of the Empty and CRC-Loaded Nanoparticles

**Figure 12:**
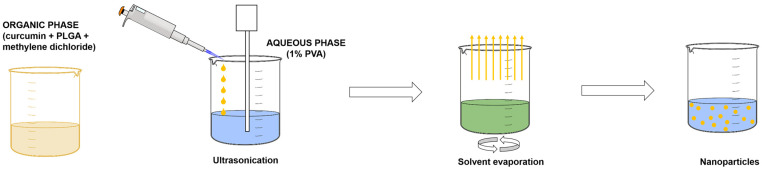
Schematic illustration of the preparation of NP (plain nanoparticles) and NP-CRC (curcumin-loaded nanoparticles) by solvent evaporation technique.

The nanoparticles were prepared via single emulsification and nanoprecipitation techniques with some modifications [10] as presented schematically in Figure 12. Briefly, the PLGA was dissolved in methylene chloride (10 mg/mL), and this organic phase was further dropwise emulsified in 1% (*w*/*v*) PVA aqueous solution under sonication (Bandelin Sonopuls HD3100, Bandelin Electronics, Berlin, Germany) over 1 min at 75% amplitude. The organic phase to water phase was set to 1:5. Methylene chloride was further evaporated under magnetic stirring (400 rpm) overnight and resulted in the preparation of the plain nanoparticles (NP). Similarly, the drug-loaded nanoparticles (NP-CRC) were prepared with curcumin being dissolved in the organic phase prior to the addition of PLGA. The thus-obtained nanoparticles were subsequently lyophilized with 5% (*w*/*v*) mannitol used as a cryoprotectant and labeled as LNP and LNP-CRC correspondingly. The lyophilization was carried out with the Alpha 3–4 LSC basic semi-industrial freeze-dryer (Martin Christ, Gefriertrocknunganlagen GmbH, Osterode, Germany). The samples were frozen at −40 °C for 24 h. Following, the first drying step was executed at −110 °C and 0.125 mbar for 72 h with a second drying at −110 °C and 0.05 mbar for 6 h. The samples were then sealed and stored for further characterization.

### 4.3. Nanoparticle Characterization

Full physicochemical characterization as well as an in vitro release study was performed for the empty and CRC-loaded nanoparticles, either in a suspension form or after lyophilization, as specified below.

#### 4.3.1. Size, Polydispersity Index (PDI), and Zeta Potential

Dynamic Light Scattering (DLS) analysis was performed immediately after the sample preparation by zeta sizer (Zeta-master, Malvern Instruments, Worcestershire, UK). It was carried out at 25 °C with a scattering angle of 90° in triplicate using water as a disperse medium. The lyophilized nanoparticles were redispersed in water, and their size, PDI, and zeta potential were evaluated immediately after the lyophilization procedure and after 6 months of storage at 4 °C. For the redispersion, distilled water was slowly added to the inner wall of the vials and the lyophilizate was allowed to wet for 10 min and then fully reconstituted by vortexing (Vortex Genius 3, IKA-Werke GmbH & Co.KG, Staufen, Germany) for 3 min.

#### 4.3.2. Fourier-Transform Infrared (FTIR) Spectroscopy

The FTIR analysis was conducted using a Thermo-Nicolet FTIR instrument (Thermo Fischer Scientific, Waltham, MA, USA) fitted with an attenuated total reflectance (ATR) device in the scope of 4000–400 cm^−1^ and at a resolution of 4 cm^−1^. The IR spectra of free ingredients and nanoparticles were captured.

#### 4.3.3. Thermogravimetric Analysis (TGA)

The thermal analyses were performed in the LABSYSEvo (SETARAM, Caluire, France) apparatus in a temperature diapason of 20–800 °C with a heating rate of 10 °C/min in Ar flow.

#### 4.3.4. Scanning Electron Microscopy (SEM)

The morphology of the nanoparticles was evaluated with the help of JSM-5510, JEOL, Japan, at 10 kV. A drop of the freshly prepared nanoparticle suspension (NP and NP-CRC) was placed on a microscope cover slide and left to evaporate. It was then coated with gold for 30 s using a sputter–coater (JSC 1200, JEOL, Tokyo, Japan) in an inert atmosphere and observed with an acceleration of 15 kV and a magnification of 10.00 k.

#### 4.3.5. Powder X-Ray Diffraction (PXRD)

Powder XRD patterns were recorded in the 10–90°2θ range at a Bragg–Brentano geometry with CuKα radiation (λ = 1.5418 Å) and a LynxEye detector in a Bruker D8 Advance diffractometer (Karlsruhe, Germany). The phase composition was determined using EVA v4 software and the ICDD-PDF2 (2021) database. The Topas-4.2 program was used to calculate the unit cell parameters of the phases.

#### 4.3.6. Encapsulation Efficiency and In Vitro Drug Release

The encapsulation efficiency (EE %) was evaluated indirectly based on the charged amount of curcumin (CRC_total_) added during the preparation and the amount found in the supernatant (CRC_free_) after centrifugation (Equation (1)). The amount of CRC was evaluated at λ = 428 nm with a spectrophotometer (Thermo Scientific Evolution 300, Madison, WI, USA) and calculated based on a beforehand prepared standard curve.

(1) $$EE\%=\frac{{CRC}_{total}-{CRC}_{free}}{{CRC}_{total}}\times 100$$

The in vitro dissolution profile of free CRC and loaded CRC (NP-CRC) was investigated in distilled water. In a soaked overnight cellulose dialysis membrane with MWCO 10,000 Da, a sample equal to 1 mg of drug (free CRC or lyophilized LNP-CRC) was placed together with 1 mL of water. The tubing was properly closed and inserted in the release medium and shaken in a thermostatic water bath at 37 ± 0.5 °C. Aliquot samples were withdrawn at predetermined time points, and the release volume was restored. The amount of CRC released was spectrophotometrically determined as discussed earlier.

In addition, the release data were fitted with zero-order, first-order, Higuchi, and Korsmeyer–Peppas release models in order to suggest the most adequate release mechanism. For this purpose, the DSolver plug-in of Excel was utilized.

### 4.4. In Vitro Cell Experiments

#### 4.4.1. Cell Line

The cell line HaCaT (originating from human keratinocytes) was purchased from Sigma Aldrich (ACACC cell lines). Cells were cultivated in DMEM culture medium, containing 10% fetal calf serum, 2 mM l-glutamine, and a high glucose concentration (4.5 g/L). Cells were constantly maintained in 75 cm^2^ flasks in an environment of 5% CO_2_ and 37 °C. When the cell layer reached approximately 80% confluence, they were trypsinized and pipetted in 96-well plates at a density of 1 × 10^4^ cells/mL. The plates were then placed in an incubator at 37 °C with 5% CO_2_ for a period of 24 h so that cells could attach to the well surface.

#### 4.4.2. Evaluation of Protective Effects on the Cell Viability of the H_2_O_2_-Induced Oxidative Stress Model

Cell viability was evaluated by means of the MTT assay. Briefly, cells were first treated with test solutions with curcumin concentrations between 0.01 and 90 μM of free (CRC) and nanoparticle-loaded curcumin (NP-CRC) and with 0.024–240 μg/mL of free nanoparticles. The principle of the assay is that NADPH-dependent oxidoreductases and dehydrogenases in live cells reduce yellow MTT to the violet formazan, which forms as insoluble intracellular crystals. The resulting formazan is dissolved in DMSO, and its quantity is determined spectrophotometrically at 570 nm using a Synergy 2 multi-plate reader. (BioTek Instruments, Inc., Highland Park, Winooski, VT, USA) [58].

The potential protective effects of CRC and NP-CRC were tested in an in vitro model of H_2_O_2_-induced toxicity on human HaCaT keratinocytes. The experimental design was as follows: cells were pretreated with CRC (0.01–20 μM) or NP-CRC at equimolar curcumin concentrations or with the empty nanoparticles (0.24–66 μg/mL) for 120 min. Then, cells were exposed to 200 μM H_2_O_2_ for 24 h, and cell viability was assessed using the MTT assay as described above [58].

#### 4.4.3. Statistical Analysis

GraphPad Prism 8 software was applied for statistical evaluation of the results. The statistical significance of comparisons between groups was evaluated using ANOVA with Dunnett’s post-test. The following alpha levels were adopted when plotting statistical significance: * *p* < 0.05; ** *p* < 0.01; and *** *p* < 0.001.

### 4.5. In Situ Hydrogel Preparation, Optimization, and Characterization

Six plain gel compositions, divided into two sets according to gelling agents’ concentration, were prepared via the two-stage cold method. The gels labeled EG1, EG2, and EG3 contained 0.1% (*w*/*v*) carbomer and 16%, 17%, and 18% (*w*/*v*) Poloxamer 407, correspondingly. The other three gels, EG4, EG5, and EG6, were based on 0.2% (*w*/*v*) carbomer and the same levels of Poloxamer 407. The composition of the investigated plain gel formulations and their coding are presented in Table 1.

Poloxamer 407 was used as a main temperature-sensitive gelling agent. In addition, carbomer was included. At the first stage, Poloxamer 407 was added to distilled water and let to fully dissolve overnight at 4 °C. Afterwards, carbomer was added, left to hydrate for 24 h, and then neutralized with 0.1 N sodium hydroxide.

The gels were then physicochemically characterized with regard to the gelation temperature, in vitro gelling time, and gel erosion. The gelation temperature was estimated based on the methodology proposed by Wei et al. [59] with slight modifications. In brief, a sample of 10 g of each of the prepared gels was placed in a low-temperature paraffin bath. A thermometer (IKA ETS-D5, IKA-Werke GmbH & Co.KG, Staufen, Germany) was used to precisely measure the temperature. The samples were gradually heated with approximately 1 °C per minute while stirred with a magnetic bar at 150 rpm (IKA RCT standard, IKA-Werke GmbH & Co.KG, Staufen, Germany). The temperature that caused a full stop of the magnetic bar was considered the gelation temperature. The experiments were performed in triplicate.

The in vitro gelling time evaluation applied the procedure proposed by Bhalerao et al. [60]. For this purpose, a fresh simulated tear fluid (STF) with pH 7.4 was prepared. The STF contained 0.67% *w*/*v* sodium chloride, 0.20% *w*/*v* sodium bicarbonate, 0.14% *w*/*v* potassium chloride, and 0.008% *w*/*v* calcium chloride dihydrate in distilled water. The final pH was adjusted with 0.1 M hydrochloric acid. All hydrogel samples were stored for 6 h at room temperature prior to the measurement. The gelling time was recorded visually after placing a drop of the investigating sample (100 µL) in a pre-heated STF (2 mL, 35 ± 0.5 °C). All tests were carried out in triplicate.

The gel erosion was evaluated in a shaking water bath on a sample of 2 g gel, which was placed in a pre-weighed vial for 10 min at 35 ± 0.5 °C. Two milliliters of pre-heated STF (35 ± 0.5 °C) were then added to each vial, followed by the placement in the shaking water bath. At specific time points, the STF was removed and the weight of the vials noted. The difference in the weight between two successive time points represented the dissolved gel. By plotting the weight of the dissolved gel as a function of time, the erosion profile was obtained. Three runs of experiments were executed, and the results are given as mean values with standard deviations [13].

### 4.6. Composite In Situ Hydrogel Preparation, Rheology, and In Vitro Permeation Study

The optimal gel selected based on the gelation temperature, gelling time, and gel erosion was applied as an in situ semisolid carrier for the prepared nanoparticles. The NP-embedded gels were prepared following the same procedure as explained earlier but with replacing the aqueous medium with the freshly prepared and filtered (0.45 µm) suspension. The free drug gel (G-CRC) and the nanocomposite gel (NP-G-CRC) were characterized for their gelation temperature, gelling time, and erosion as explained prior.

Furthermore, the free drug gel (G-CRC) and NP-CRC-embedded gel (NP-G-CRC) were characterized in terms of rheology with a rheometer, HAAKE RheoStress 600 (Thermo Scientific, Karlsruhe, Germany). Dynamic rheological measurements were conducted in controlled deformation mode with a parallel plate sensor system (top plate diameter = 20 mm; gap = 1 mm) in triplicate at 20 °C and 35 °C. The oscillation amplitude sweep tests were performed at a frequency of 1 Hz in the ɣ_0_ range from 0.001 to 100. The frequency sweep tests were performed in the range of 0.1–10 Hz at ɣ_0_ = 0.01.

Both gels (NP-G-CRC and G-CRC) were used in an in vitro permeation test. An automated Franz-diffusion system (Logan system 913-6; Logan Instruments Corp., 19C Schoolhouse, Somerset, NJ, USA) was applied. As a disperse medium, 12 mL of freshly prepared STF at 35 ± 0.5 °C was utilized. The donor compartment was filled with 2 g of the corresponding thermoset gel. A dialysis membrane (Spectra/Por^®^ cellulose, MWCO 10000, Carl Roth GmbH, Karlsruhe, Germany) with an effective area of 1.54 cm^2^ was used to separate the two compartments. Aliquot samples were automatically withdrawn at specific time points, and the fresh medium was replenished. The amount of released curcumin was spectrophotometrically determined, and it was used to calculate the flux according to the following equation:

(2) $$Q_{CRC}=\frac{C_{n}.V_{A}+\sum_{i}^{n-1}C_{i}.V_{s}}{S}$$

where Q_CRC_ represents the cumulative curcumin amount penetrated through the membrane; C_n_—curcumin concentration in the nth sample; C_i_—curcumin concentration in the ith sample; V_A_—acceptor medium volume (12 mL); Vs—sample volume (1 mL); and S—effective permeation surface area of the membrane (1.54 cm^2^). The steady-state flux (J_ss_) was determined by the slope of the linear part of the plotted curve [61,62].

## Figures and Tables

**Table 1:** Size distribution, zeta potential, and polydispersity index of the plain nanoparticles (NP) and the curcumin-loaded nanoparticles (NP-CRC) and the corresponding lyophilized samples (LNP and LNP-CRC) upon their preparation and after six months of storage.

Nanoparticles	After Preparation			After 6 Months of Storage		
	Size, nm	PDI	Zeta, mV	Size, nm	PDI	Zeta, mV
NP (in suspension)	323.1 ± 1.3	0.216	−15.6 ± 2.2	390.5 ± 5.2	0.337	−4.75 ± 0.88
NP-CRC (in suspension)	296.4 ± 3.1	0.172	−7.1 ± 0.8	424.4 ± 1.6	0.290	0.35 ± 0.09
LNP (lyophilized)	220.3 ± 1.8	0.196	−10.65 ± 2.5	223.1 ± 0.4	0.222	−8.55 ± 3.37
LNP-CRC (lyophilized)	127.4 ± 2.3	0.168	−8.73 ± 2.8	125.5 ± 3.5	0.230	−3.57 ± 0.15

**Table 2:** Composition and coding of the investigated in situ hydrogels (gelation temperature, gelling time upon simulated tear fluid (STF) dilution, and the mean of three replicates ± SD).

Formulation	Poloxamer 407, % *w*/*v*	Carbomer, % *w*/*v*	Gelation Temperature, °C	Gelling Time Upon STF Dilution, s
EG1	16	0.1	32.30 ± 0.40	9.57 ± 0.64
EG2	17	0.1	31.40 ± 0.36	8.99 ± 0.28
EG3	18	0.1	28.13 ± 0.31	5.75 ± 0.11
EG4	16	0.2	31.36 ± 0.50	6.82 ± 0.66
EG5	17	0.2	28.87 ± 0.25	3.87 ± 0.45
EG6	18	0.2	26.27 ± 0.45	2.96 ± 0.36

EG = empty gel; STF = simulated tear fluid.

**Table 3:** Rheology and gelation characteristics of the investigated gels.

Hydrogel Formulation	Viscosity, Pa.s	G’, Pa	G’’, Pa	Gelation Temperature, °C	Gelling Time Upon STF Dilution, s
G-CRC	0.244 ± 0.01 (20 °C, shear rate >14 s^−1^)	-	-	31.77 ± 0.52	9.05 ± 0.33
	1292 ± 33 (35 °C)	8050 ± 146	1059 ± 103		
NP-G-CRC	1.578 ± 0.35 (20 °C, shear rate >14 s^−1^)	-	-	30.91 ± 0.31	8.67 ± 0.54
	2129 ± 24 (35 °C)	13,300 ± 246	1451 ± 96		

## Data Availability

Original data are contained within this article.
